# Laparoscopic Appendectomy for Left-Sided Acute Appendicitis in Situs Inversus Totalis: A Case Report and Technical Considerations

**DOI:** 10.7759/cureus.109343

**Published:** 2026-05-21

**Authors:** Ahmad E Al-Mulla, Omar Shalaby, Mohamed N Mohamed

**Affiliations:** 1 Department of General Surgery, Farwaniya Hospital, Kuwait City, KWT; 2 Department of Surgery, Farwaniya Hospital, Kuwait City, KWT

**Keywords:** appendicitis, congenital abnormalities, dextrocardia, laparoscopic appendectomy, left iliac fossa pain, situs inversus totalis

## Abstract

Left-sided acute appendicitis is an exceptionally uncommon presentation, most often associated with congenital anomalies such as situs inversus totalis (SIT), and may contribute to diagnostic delays. A 31-year-old man presented with a two-day history of migratory abdominal pain localized to the left lower quadrant, with nausea, vomiting, and anorexia. He was hemodynamically stable without leukocytosis. Contrast-enhanced CT demonstrated SIT and an inflamed appendix in the left lower quadrant (approximately 11 mm), with periappendiceal fat stranding and a small volume of free fluid, without pneumoperitoneum. Laparoscopic appendectomy was performed using modified team positioning and port placement to accommodate the mirrored anatomy. Recovery was uncomplicated, and histopathology confirmed acute appendicitis with periappendicitis and no malignancy. Appendicitis should be considered in the differential diagnosis of left lower-quadrant pain; CT supports timely diagnosis in SIT, and laparoscopy is safe and effective when anatomic and ergonomic factors are anticipated.

## Introduction

Acute appendicitis is among the most common surgical emergencies and typically presents with periumbilical pain that migrates to the right lower quadrant. In routine practice, diagnosis is supported by the clinical picture, inflammatory markers, and imaging; CT offers high diagnostic accuracy when the presentation is atypical. Given the variable anatomical position of the appendix, a considerable proportion of patients present with pain outside the right lower quadrant; this atypical distribution may obscure the diagnosis, delay definitive intervention, and increase the risk of perforation and intra-abdominal sepsis [1].

Left-sided acute appendicitis is rare and is most often associated with congenital anatomic variants, particularly situs inversus totalis (SIT) and midgut malrotation [1-3]. SIT is characterized by a mirror-image arrangement of the thoracic and abdominal viscera and has been reported at frequencies ranging from approximately one in 10,000 to one in 50,000 live births [4]. When appendicitis occurs in SIT or malrotation, patients may present with left lower quadrant pain and an atypical abdominal examination, frequently leading to initial misdiagnosis as diverticulitis, ureteric colic, or colitis and thereby delaying definitive management [3,5]. In addition, incomplete transposition of visceral pain pathways has been described, which can further confound symptom localization [3,6].

Cross-sectional imaging is, therefore, pivotal in patients with nonclassical features or discordant physical findings. CT can simultaneously confirm appendiceal inflammation, define its anatomic location, and identify rotational anomalies, allowing prompt operative planning [1-3]. Laparoscopy is particularly advantageous in this setting because it can establish the diagnosis when uncertainty remains and permits a safe appendectomy with tailored operating room ergonomics and port placement to accommodate mirrored anatomy [3,7,8]. Technical reports and case series emphasize that anticipating right-left disorientation, optimizing instrument angles, and planning team positioning can mitigate operative difficulty and support favorable outcomes [1,3,7,8].

This report describes a case of acute left-sided appendicitis in a patient with SIT that was successfully managed with laparoscopic appendectomy, with particular emphasis on the radiologic features and operative strategy.

## Case presentation

A 31-year-old man with no significant past medical history presented to the emergency department at Farwaniya Hospital with a two-day history of left-sided abdominal pain associated with nausea, multiple episodes of vomiting, and anorexia. He denied having a fever and constipation. The pain began in the periumbilical region and later localized to the left lower quadrant. On examination, he was hemodynamically stable and afebrile. Abdominal examination revealed tenderness and guarding in the left lower quadrant. Laboratory investigations on presentation (Table 1) showed no leukocytosis, marked neutrophilia, elevated CRP, hemoglobin within the reference range, and a mildly elevated platelet count. Given the unusual left-sided localization, the initial differential diagnosis included diverticulitis and ureteric colic; however, the migratory pain pattern and inflammatory markers prompted contrast-enhanced CT to clarify the diagnosis and evaluate for an underlying anatomic anomaly.

**Table 1:** Laboratory investigations on presentation

Parameter	Result	Reference range	Interpretation
WBC count	9.3 × 10^9^/L	4.0–11.0 × 10^9^/L	Normal
Neutrophils	90%	40-75%	Marked neutrophilia
CRP	140 mg/L	0-5 mg/L	Marked inflammatory response
Hemoglobin	13.5 g/dL	13.0-17.0 g/dL	Normal
Platelets	456 × 10^9^/L	150-400 × 10^9^/L	Mild reactive thrombocytosis

**Figure 1:**
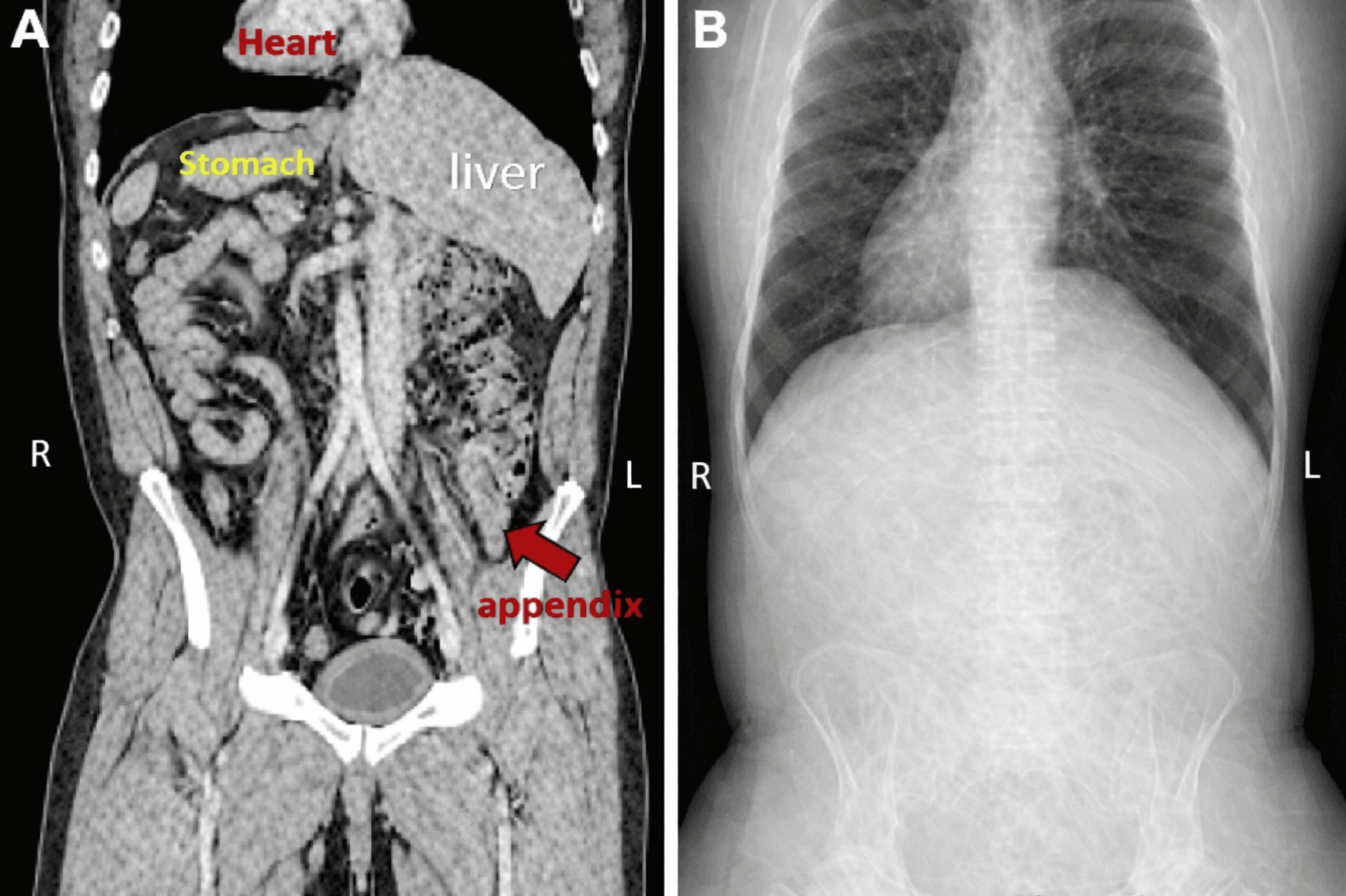
(A) Coronal abdominal CT image demonstrating the cecum and appendix on the left side. (B) Chest radiograph demonstrating a right-sided cardiac apex (dextrocardia).

Abdominal CT scan demonstrated SIT and an inflamed appendix in the left lower quadrant, measuring approximately 11 mm in diameter, with mural thickening and hyperenhancement. Additional findings included periappendiceal fat stranding and a small volume of free fluid. The cecum showed mild reactive mural thickening and edema, along with multiple enlarged reactive lymph nodes. No pneumoperitoneum was identified (Figure 1A, 1B).

The patient was informed about the results and surgical options, with laparoscopic appendectomy suggested as the best option. Written consent was obtained. After evaluation by the anesthesiology team, the patient was deemed fit for surgery.

Surgical technique

**Figure 2:**
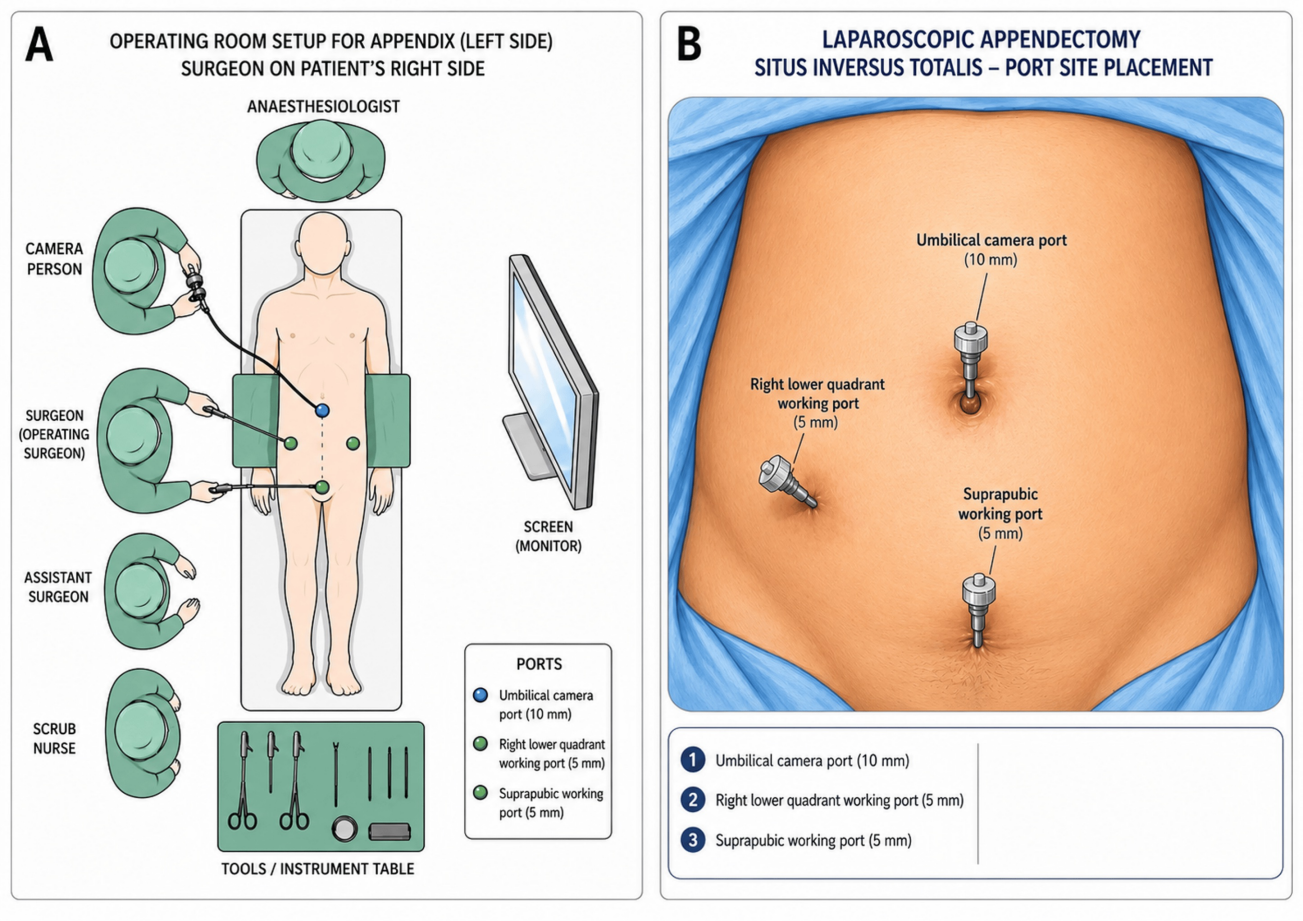
(A) Operating team positioning during laparoscopic appendectomy in SIT. (B) Port positioning used for the procedure. SIT, situs inversus totalis This figure was created using Adobe Photoshop (Adobe Inc., San Jose, CA, USA) and Microsoft PowerPoint (Microsoft Corporation, Redmond, WA, USA).

The patient was placed supine. The surgeon and assistant stood on the patient’s right side. Pneumoperitoneum was established using a Veress needle inserted in the right upper quadrant. A 10-mm umbilical port was inserted (using a visual port) for the camera, and two 5-mm working ports were placed in the right lower quadrant and suprapubic region (Figure 2A, 2B).

The cecum and appendix were identified in the left lower quadrant, with minimal free fluid. The appendix was inflamed, with a thickened mesoappendix and a healthy base. The liver and gallbladder were visualized in mirrored positions.

Laparoscopic appendectomy was performed by dissecting the mesoappendix with a harmonic scalpel. The appendiceal base was ligated with an Endoloop, and the specimen was retrieved using a 10-mm endobag. All trocars were removed under direct vision, and the 10-mm port-site fascia was closed with Vicryl 1-0.

Outcome and follow-up

Postoperatively, the patient received three doses of IV piperacillin/tazobactam 4.5 g. Metronidazole 500 mg was also administered in accordance with the local institutional protocol, although piperacillin/tazobactam provided the primary broad-spectrum and anaerobic coverage in this case. By postoperative day 1, he was mobilizing and passing flatus. Oral fluids were initiated, and his diet was advanced gradually to a regular diet as tolerated. He was discharged on postoperative day 2 after tolerating oral intake and passing a bowel movement; the wounds were clean.

**Figure 3:**
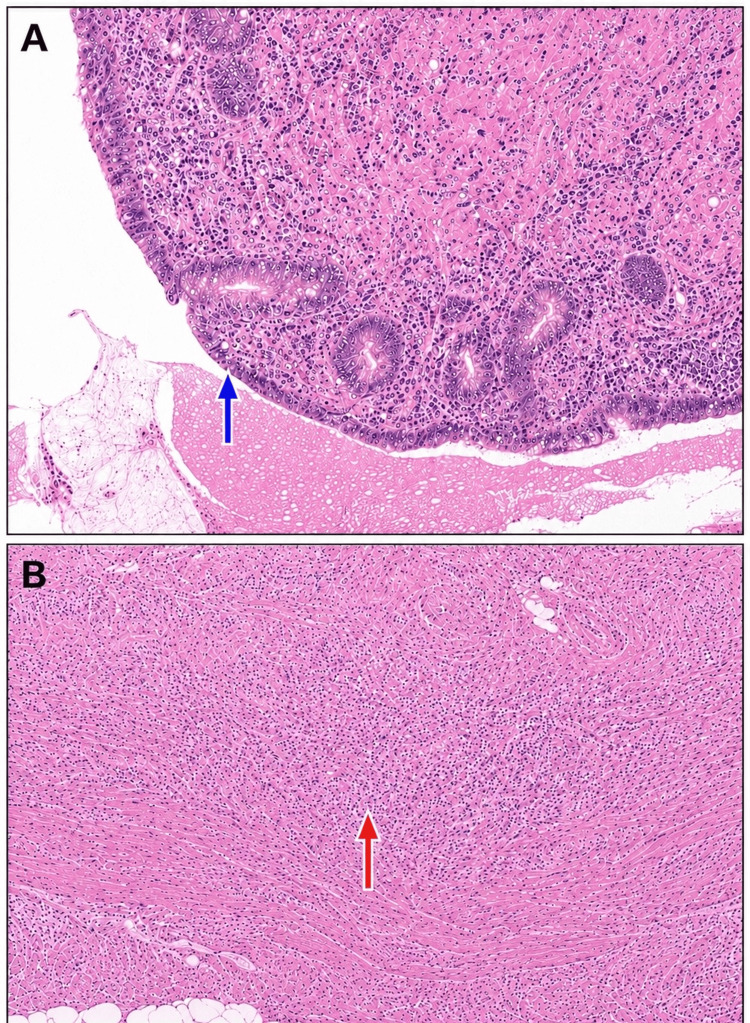
Histopathological findings in the resected appendix. (A) Hematoxylin and eosin section showing acute mucosal and submucosal inflammation. (B) Acute inflammatory infiltrate extending into the muscularis propria, consistent with acute appendicitis.

At the 14-day postoperative review in the surgical outpatient clinic, the patient remained asymptomatic, the wounds were healing satisfactorily, and he had resumed normal activity. Histopathological examination confirmed acute appendicitis with periappendicitis, with no evidence of malignancy. No further follow-up was considered necessary (Figure 3A, 3B).

## Discussion

Left lower quadrant pain presents a broad differential diagnosis encompassing sigmoid diverticulitis, ureteric colic, colitis, and, in women, gynecological causes. Previous series have demonstrated that patients with left lower quadrant pain may exhibit associated features, including rebound tenderness, palpable mass, nausea or vomiting, and fever [5].

Acute appendicitis is a common surgical emergency; however, left-sided appendicitis is rare and is most often associated with congenital anomalies such as SIT or midgut malrotation [1]. The incidence of appendicitis in patients with SIT is low. This atypical presentation may delay diagnosis and increase the risk of complications, including perforation, abscess formation, inflammatory mass, and gangrene [3].

Clinicians should consider appendicitis in cases of migratory and focal pain, even when tenderness is localized to the left lower quadrant. Left-sided acute appendicitis poses a diagnostic challenge, particularly in the absence of prior knowledge of congenital anomalies such as SIT. Although visceral organs may be transposed, the nervous system does not always display corresponding transposition, as reported in 14.7-31% of patients with SIT or midgut malrotation [3,6]. In the present case, the absence of leukocytosis despite marked neutrophilia and elevated CRP highlights that the inflammatory profile may still support a diagnosis of appendicitis even when the total white blood cell count remains within the reference range. Given the unreliability of symptoms alone, imaging is essential for accurate diagnosis.

Plain radiography may provide helpful clues; in the present case, dextrocardia and a gastric bubble on the right side suggested the underlying condition. Although ultrasound is utilized in the diagnosis of appendicitis, its accuracy is operator dependent and may be reduced in obese patients or those with excessive bowel gas. IV contrast-enhanced CT of the abdomen remains the most accurate and reliable diagnostic modality, with a reported accuracy of 90-98% [9], and is the current standard in most emergency settings. In the absence of CT or in cases of ongoing diagnostic uncertainty, laparoscopic exploration is a reasonable alternative.

Beyond left-sided presentations, atypical appendiceal locations can produce misleading pain patterns, including epigastric pain, underscoring the importance of maintaining diagnostic vigilance [10,11].

Laparoscopic appendectomy in patients with SIT and midgut malrotation is safe and effective [8]. It reduces the risk of wound infection, improves visualization, particularly in cases involving additional abdominal pathology, and facilitates faster postoperative recovery. In this case, the patient was mobilizing by postoperative day 1, was discharged on postoperative day 2, and had returned to normal activity by 14 days postoperatively.

Operative considerations

Preoperative imaging should be reviewed to confirm mirrored anatomy. An initial diagnostic laparoscopy should then be performed to reorient key landmarks, such as the cecum, ileocecal valve, and appendix. The operating team’s position and port placement should be adjusted to maintain comfortable instrument angles. Options include a mirror-image setup or right-sided working ports with the team positioned on the patient’s right, as in this case. Ergonomic challenges may include right-left disorientation, cross-handed manipulation, and instrument clashing, particularly during mesoappendix dissection and appendiceal base control; these difficulties can be reduced by thoughtful port spacing, deliberate stepwise dissection, and early adjustment of operator position when needed.

When anatomy is uncertain, alternative access sites for pneumoperitoneum, such as the right upper quadrant, can reduce the risk of injury. During mesoappendix dissection and base control, deliberate, stepwise identification helps mitigate right-left disorientation and avoid misidentifying structures. Port placement should be modified intraoperatively if exposure is suboptimal.

## Conclusions

Left-sided appendicitis in patients with SIT should remain an important consideration in the differential diagnosis of left-sided abdominal pain. Timely diagnosis depends on integrating clinical assessment with appropriate imaging. Laparoscopic appendectomy is a safe and feasible approach in SIT, provided that mirrored anatomy is recognized preoperatively and technical modifications in team positioning, port placement, and intraoperative orientation are made accordingly.
